# Postmenopausal Tuberculosis Endometritis

**DOI:** 10.1155/2007/27028

**Published:** 2007-05-08

**Authors:** Kemal Güngördük, Volkan Ulker, Ahmet Sahbaz, Cemal Ark, Alı Ismet Tekırdag

**Affiliations:** Department of Obstetrics and Gynecology, Gynecological Clinic, Istanbul Bakirkoy Women and Children Hospital, Istanbul, Turkey

## Abstract

Tuberculosis remains a global health problem, primarily in developing countries with inadequate health services. A significant portion of tuberculosis in these settings is extrapulmonary, including tuberculosis of the genitourinary tract. Patients with genital tuberculosis are usually young women detected during work up for infertility. After menopause, tuberculosis of the endometrium is a rare possibility probably because of the decreased vascularity of the tissues. We present a case of endometrial tuberculosis with postmenopausal vaginal bleeding.

## 1. INTRODUCTION

Morgani was the first to describe genital tuberculosis in the mid-eighteenth century and *Tuberculous bacillus* was discovered in 1882 by Koch [1]. Tuberculosis (TB) of the genital tract is almost invariably secondary to disease elsewhere, usually in the lungs. Five to 13% of patients with pulmonary TB develop genital infection [1]. Female genital TB is a rare disease in some developed countries, but it is a frequent cause of chronic pelvic inflammatory disease (PID) and infertility in other parts of the world [2].

However, World Health Organization reported that tuberculosis was a global emergency in the early 1990s because of the increased number of HIV-infected patients, increased number of immigrants to the industrialized countries from developing countries, and various social problems such as poverty and homelessness [3].

The HIV pandemic poses major problems for the tuberculosis control program and for the individual clinicians treating HIV-related tuberculosis. HIV-related immunosuppression is the single most potent risk factor for progression from latent tuberculosis infection to active tuberculosis. As a result, HIV is a major factor driving the global resurgence of tuberculosis; incidence rates of tuberculosis in countries with high prevalence of HIV infection have increased up to five-fold [4].

The disease is responsible for 5% of all female pelvic infections and occurs in 10% cases of pulmonary tuberculosis. Although most of the affected belong to reproductive age group, the disease has been reported in postmenopausal females as well [5].

## 2. CASE

A 55-year-old postmenopausal woman, gravida 5 para 5, menopausal for 3 years presented with irregular vaginal bleeding for the past one month associated with passage of clots. There was no pain at abdomen, foul smelling discharge, fever, loss of weight, loss of appetite, or prior postcoital bleeding. The patient was otherwise well and had no significant medical history. She had no personal or family history of gynecological or other malignancy. She was not smoking or had alcohol and substance abuse. She has never used oral contraceptives or HRT. General physical and systemic examination detected no abnormality. On *per speculum* examination vagina was healthy and there was some bleeding from the cervix. Uterus was anteverted, 8–10 weeks in size and bilateral fornices free. Pap smear could not be performed because of vaginal bleeding. A Pap smear performed in another clinic four months ago was normal.

**Figure 1:**
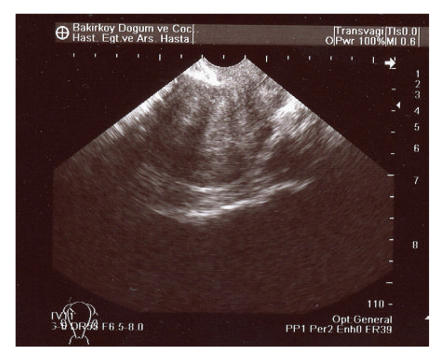
Transvaginal pelvic ultrasound showed an abnormal endometrial echo pattern. The endometrium was heterogenous and had irregular surface.

**Figure 2:**
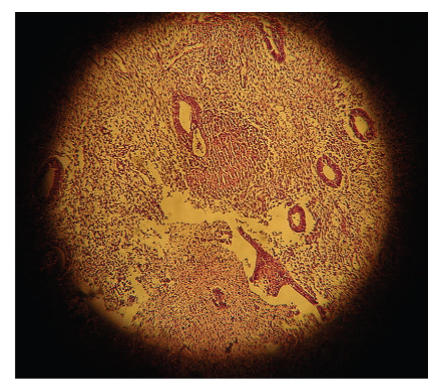
A classical tuberculosus granuloma formed by epithelioid macrophages, cuffed by lymhocytes and containing a Langhans giant cell (H&E×40).

Transvaginal pelvic ultrasound was performed and showed an anteverted uterus with a grossly abnormal endometrial echo pattern. The endometrium was heterogenous and had an irregular surface (Figure 1). After the history and physical examination, endometrial biopsy was performed with profuse, curettings obtained. Blood loss was not excessive and the patient was discharged home the same day. Histopathology of the curettings showed epithelioid cell granulomas with Langhans giant cells (Figure 2).

The patient's hepatitis and HIV status were negative. All hematological and biochemical investigations were normal. PCR was performed on the formalin-fixed paraffin-embedded tissue for diagnosis of tuberculosis. PCR was positive for *Mycobacterium tuberculosis* in the endometrium. Other tests for tuberculosis, namely, Mantoux was positive at 32mm and ESR was 77mm. A chest X-ray showed mild cardiomegaly but no evidence of pulmonary tuberculosis. The patient received antituberculosis treatment (4 drugs: isoniazid, rifampicin, ethambutol, and pyrazinamide) for six months and it has been learned that her bleeding ended in the fourth week of her treatment. At subsequent follow-up visits, patient is doing well and has been disease-free for 10 months.

## 3. DISCUSSION

Genital TB infection is usually caused by reactivation of organisms from systemic distribution of *Mycobacterium tuberculosis* during primary infection. It is estimated that about 8 million new cases of tuberculosis infection occur each year worldwide, and 95% of these cases are in undeveloped countries. Tuberculosis primarily affects the lungs, but about one third of the patients also have involvement of extrapulmonary organs such as the meninges, bones, skin, joints, genitourinary tract, and abdominal cavity. Extrapulmonary tuberculosis represents a progressively greater proportion of new cases in the developed countries, and this trend is still increasing [6, 7]. The resurgence of TB in the developed world may be explained by the increased numbers of immigrants from areas where this disease is endemic as well as the increasing prevalence of HIV infection with TB being one of the opportunistic infections in individuals infected with this virus [1]. People with latent tuberculosis infection are at higher risk of progression to active disease if coinfected with HIV, with the risk increasing to over 20 times that in HIV-negative people as immunosuppression worsens. HIV infection also causes recent tuberculosis infection to progress more rapidly to disease [7, 8].

Direct transmission between sexual partners has been documented. Spread from other intraperitoneal foci is rare [7]. The fallopian tubes are affected in almost 100% of the cases followed by the endometrium in 50%, ovaries in 20%, cervix in 5%, and vagina and vulva in <1% [1, 8]. Tuberculous endometritis is almost invariably associated with tuberculous salpingitis except in the postmenopausal women, in whom there may be no tubal disease [8].

Patients with genital tuberculosis are usually young women during work up for infertility. Genital tuberculosis is rare in postmenopausal women and responsible for only approximately 1% of postmenopausal bleeding. The endometrium is affected in 60–70%. The low incidence in the postmenopausal age group is difficult to explain. Most authors believe that an atrophic endometrium offers a poor milieu for the growth of mycobacterium [9]. In a western study of 475 cases of postmenopausal bleeding, no case of tuberculosis was reported, while Indian studies report that in women with genital tuberculosis, 1%–1.6% present with postmenopausal bleeding [10].

Genital TB tends to be an indolent infection, disease may not manifest for years after initial seeding. The most common presentations reported were infertility (44%), pelvic pain (25%), vaginal bleeding (18%), amenorrhea (5%), vaginal discharge (4%), and postmenopausal bleeding (2%). Less common presentations included abdominal mass, ascites, tuboovarian abscess, and vague abdominal distention [11].

In a recent report of primary endometrioid adenocarcinoma coexisting with endometrial tuberculosis and tubal adenocarcinoma coexisting with genital tuberculosis, the authors concluded that although the two entities are extremely rare, occurrence in regions with a high prevalence of tuberculosis may not be uncommon [12].

Genital tract TB may be suspected from the medical history, including abnormal test results such as high sedimentation rate, falsely elevated CA-125 level, and chest X-ray film with lesions suggestive of TB. On pelvic examination of patients, Saracoglu and colleagues reported normal physical examination results (43%), adnexal mass (23.6%), myoma-like lesion (23.6%), adnexal tenderness (4.2%), irregular uterus (1.4%), uterine prolapse (1.4%), and cervical polyp (1.4%) [13]. The Mantoux test (tuberculin skin test) showed a sensitivity of 55% with a specificity of 80% in female genital TB [14]. Saracoglu and colleagues found that more than 75% of patients with genital TB had a normal chest X-ray film. On pelvic imaging (including hysterosalpingogram) of patients, coronal block, fimbrial block, beaded tube, hydrosalpinx, and/or filling defects in the uterine cavity were present in more than 70% [12]. Adnexal mass, thickened omentum, fluid in the pelvic cavity, and adhesions have been demonstrated on pelvic ultrasound. Molecular testing, with polymerase chain reaction and DNA hybridization, has been used in the presumptive diagnosis of genital TB [14]. The diagnosis of the disease is difficult. Histopathological evidence in biopsy of premenstrual endometrial tissue or demonstration of tubercle bacilli in culture of menstrual blood or endometrial currettings only can provide the certain diagnosis of disease. The typical lesions in genital TB are epithelioid cell granulomas with or without Langerhans giant cells. Caseation necrosis is rare and tends to be a late feature [13].

There are scant prospective data on optimal medical management of genital TB. Treatment guidelines recommend 6 months of treatment for female genital TB, providing that pyrazinamide is included for the first 2 months of treatment and that the organism is susceptible [15]. Surgical therapy usually consists of total abdominal hysterectomy and bilateral salpingo-oophorectomy. Indications for surgery include persistence of pelvic mass and recurrence of pain or bleeding after 9 months of treatment. Surgery should be performed at least 6 weeks after initiation of anti-TB therapy, because antimicrobial treatment facilitates the surgical procedure and reduces the risk of perioperative complications [16].

In conclusion, genital tuberculosis is rare in postmenopausal women and responsible for only approximately 1% of postmenopausal bleeding patients. We recommend that all patients with a positive Mantoux test result and menstrual abnormalities undergo aggressive evaluation for genital TB.
